# A novel framework for validating and applying standardized small area measurement strategies

**DOI:** 10.1186/1478-7954-8-26

**Published:** 2010-09-29

**Authors:** Tanja Srebotnjak, Ali H Mokdad, Christopher JL Murray

**Affiliations:** 1 Institute for Health Metrics and Evaluation, University of Washington, 2301 5th Ave, Suite 600, Seattle, WA 98121, USA

## Abstract

**Background:**

Local measurements of health behaviors, diseases, and use of health services are critical inputs into local, state, and national decision-making. Small area measurement methods can deliver more precise and accurate local-level information than direct estimates from surveys or administrative records, where sample sizes are often too small to yield acceptable standard errors. However, small area measurement requires careful validation using approaches other than conventional statistical methods such as in-sample or cross-validation methods because they do not solve the problem of validating estimates in data-sparse domains.

**Methods:**

A new general framework for small area estimation and validation is developed and applied to estimate Type 2 diabetes prevalence in US counties using data from the Behavioral Risk Factor Surveillance System (BRFSS). The framework combines the three conventional approaches to small area measurement: (1) pooling data across time by combining multiple survey years; (2) exploiting spatial correlation by including a spatial component; and (3) utilizing structured relationships between the outcome variable and domain-specific covariates to define four increasingly complex model types - coined the Naive, Geospatial, Covariate, and Full models. The validation framework uses direct estimates of prevalence in large domains as the gold standard and compares model estimates against it using (i) all available observations for the large domains and (ii) systematically reduced sample sizes obtained through random sampling with replacement. At each sampling level, the model is rerun repeatedly, and the validity of the model estimates from the four model types is then determined by calculating the (average) concordance correlation coefficient (CCC) and (average) root mean squared error (RMSE) against the gold standard. The CCC is closely related to the intraclass correlation coefficient and can be used when the units are organized in groups and when it is of interest to measure the agreement between units in the same group (e.g., counties). The RMSE is often used to measure the differences between values predicted by a model or an estimator and the actually observed values. It is a useful measure to capture the precision of the model or estimator.

**Results:**

All model types have substantially higher CCC and lower RMSE than the direct, single-year BRFSS estimates. In addition, the inclusion of relevant domain-specific covariates generally improves predictive validity, especially at small sample sizes, and their leverage can be equivalent to a five- to tenfold increase in sample size.

**Conclusions:**

Small area estimation of important health outcomes and risk factors can be improved using a systematic modeling and validation framework, which consistently outperformed single-year direct survey estimates and demonstrated the potential leverage of including relevant domain-specific covariates compared to pure measurement models. The proposed validation strategy can be applied to other disease outcomes and risk factors in the US as well as to resource-scarce situations, including low-income countries. These estimates are needed by public health officials to identify at-risk groups, to design targeted prevention and intervention programs, and to monitor and evaluate results over time.

## Background

There is no shortage of health-related information in the US. However, the large number of surveys and administrative systems that collect health information at the national level stands in contrast to the relative scarcity of accurate and precise local-level measurements. For example, national data sources such as the National Health and Nutrition Examination Survey (NHANES) and the National Health Interview Survey (NHIS) do not provide measurements for counties or even states. The Behavioral Risk Factor Surveillance System (BRFSS), with a sample size of more than 414,000 in 2008, is the world's largest ongoing national telephone survey. Even though the survey collects data in nearly all US counties, measurements of leading health outcomes and risk factors at the county level are not routinely produced due to small sample sizes in the majority of counties, although the CDC has produced county-level diabetes prevalence estimates since 2004 with most recent estimates for 2007. For example, in 2008, more than 80% of counties had sample sizes of less than 100. Some states purchase enhanced BRFSS samples to generate local measurements, demonstrating demand for this type of information, but for the majority of counties, these measurements are not available. The BRFSS Selected Metropolitan/Micropolitan Area Risk Trends (SMART) project analyzes selected risk factors for Metro- and Micropolitan Statistical Areas (MMSAs) with more than 500 respondents to identify the status and trends of important health problems at the local level. However, out of 3,141 US counties, only 177 MMSAs were SMART counties in 2008. On the other hand, some projects, such as the County Health Rankings [1,2], have used sparse data from a single year to directly report on and compare counties, despite the risks of drawing inaccurate inferences.

Small area measurement methods refer to a suite of statistical methods aimed at filling the need for better local information. The main procedures include direct domain estimation, indirect domain estimation, and small area modeling. Direct domain estimation uses available sample units in the domain to estimate the quantity of interest, leading to unacceptably large standard errors for small domains. Indirect estimation implicitly makes assumptions about how domains are related in time and/or space to increase the effective sample size for small domains [3]. Indirect domain estimation includes: synthetic estimators (i.e., using a reliable estimator for a large domain to derive an estimator for the small domain contained within the large domain under the assumption that the small domain has the same characteristics as the large domain); composite estimators (i.e., weighted averages of sample estimates for the same domain but from different surveys); and James-Stein estimators (also called shrinkage estimators because they shrink the mean squared error, sometimes also used in conjunction with the direct estimator in so-called "limited translation estimators"). In contrast to indirect domain estimation, small area modeling is explicit about the assumptions of relatedness in space and/or time and has variably used three strategies to deal with the limited availability of survey and administrative data: pooling data over several years [4,5]; borrowing strength in space by exploiting spatial correlations [6]; and using structured relationships with covariates to predict the quantity of interest [7]. Few studies, however, have used all three approaches in a consistent fashion at a national level. Li et al [8,9] used mixed-effects models to estimate obesity and smoking prevalence in 398 communities in Massachusetts using 1999-2005 BRFSS data. Elliott and Davis [10] used a dual-frame estimation approach to link NHIS and BRFSS data for estimating adult male tobacco prevalence in 584 counties in 1999-2000. Small area statistical methods have been used in several studies, including one nationwide assessment of diabetes by the CDC [7-9,11] and vaccination coverage monitoring during the 2004-05 influenza season in the US [12]. Recently, Caldwell et al [13] used a Bayesian multilevel approach to estimate 2005 county-level diabetes prevalence for the population 20 years and older, pooling 2004-2006 BRFSS data and the county's posterior rank distribution to identify counties with high or low diagnosed diabetes burdens. They used design-based direct estimates for 232 large counties to assess the validity of the model prevalence estimates. Congdon and Lloyd [14] applied a binary person-level random effects regression model using individual risk factors from the 2005 BRFSS and small area characteristics for 32,000 ZIP code tabulation areas. Spatial information is incorporated at the state level. But standardized methods have otherwise not been articulated, validated, or widely applied to health behavior measurements in the US.

The main limitation of small area methods for local health measurement has been the difficulty in validating a particular approach for a given health problem. Standard approaches such as in-sample fit statistics and cross validation are not useful in a small area setting as they do not adequately answer the question of how well these methods work compared to undertaking a large sample survey in each locality. Within the limitations of in-sample data, a variety of authors have explored the theory and metrics for validating model estimates [15,16]. Nevertheless, the ultimate test of predictive validity, i.e., comparing the results against the de facto gold standard, has rarely been implemented for small area measurement in public health.

In this paper, a standardized approach to small area measurement is proposed that uses all three traditions: using data from several years, exploiting spatial correlation using estimates from neighboring counties, and using structured relationships with area-level covariates to inform estimates. The critical innovation is that we create a validation environment in which the most appropriate measurement strategy can be selected and tailored to the data and variable under study. This approach is illustrated by estimating Type 2 diabetes prevalence for all counties in the US for 2008 from the 2000-2008 BRFSS.

## Methods

### Four Families of Statistical Models

The framework rests on four types of models that can, in principle, be applied to any small area measurement task. Because many determinants and patterns may be different for males and females, we modeled each sex separately, but they could be modeled jointly where appropriate. The four types are the **Naïve**, the **Geospatial**, the **Covariate**, and the **Full **model (cf. Table 1). The Naïve and Geospatial models only include individual age group (AGE_ij_) and an optional fixed effect for race group (RACE_ij_) and can thus be viewed as measurement models. They do not require additional covariate data, the availability of which may be limited or impacted by cost factors. The Covariate and Full models draw leverage from the relationships between outcome variable and additional domain-level covariates (denoted by Z_i _in model equations 1-4). All models include a random county intercept (δ_i_) with an independent variance-covariance structure, i.e., with all variances estimated and covariances assumed to be zero. Spatial relationships are not specifically incorporated into the variance-covariance structure of the random intercepts. This is accomplished through an additional covariate in the Geospatial and Full models. We also tested for random coefficients on survey year (YEAR_ij_) to allow for heterogeneous temporal patterns across counties using an independent variance-covariance structure but without improving the model fit. Lastly, the Geospatial and Full models harness spatial patterns through an additional covariate (${\bar{\delta }}_{i}^{post}$), calculated by averaging the estimated county random intercept for neighboring counties from the Naive and Covariate models, respectively. Neighboring counties are those counties that have a common boundary. For island counties, no neighbors were defined.

**Table 1:** The four model types and their respective specifications.

Model Family	Individual Race	Coefficient on Time	Spatial covariate	County-level covariates
Naive	Excluded	Fixed	No	No
	Excluded	Random	No	No
	Included	Fixed	No	No
	Included	Random	No	No

Geospatial	Excluded	Fixed	Yes, from naive model	No
	Excluded	Random	Yes, from naive model	No
	Included	Fixed	Yes, from naive model	No
	Included	Random	Yes, from naive model	No

Covariate	Excluded	Fixed	No	Yes
	Excluded	Random	No	Yes
	Included	Fixed	No	Yes
	Included	Random	No	Yes

Full	Excluded	Fixed	Yes, from covariate model	Yes
	Excluded	Random	Yes, from covariate model	Yes
	Included	Fixed	Yes, from covariate model	Yes
	Included	Random	Yes, from covariate model	Yes

Note: Sixteen models for men and women, respectively, were estimated and evaluated for (a) all available observations, (b) by sampling 100, 50, and 10 observations per county-year for counties with at least 900 respondents in 1996-2004. The sampling was repeated 10 times at each sampling level.

Thus, the basic model is a generalized linear mixed-effects regression model with binomial outcome (Y) and logit link function. The combinations of the four model families and four model specifications generate a total of 16 models for each sex, which can be summarized as follows:

Naïve model:

$$\log\frac{\mathrm{Pr}(Y_{ij}=1|sex_{ij}=k)}{1-\mathrm{Pr}(Y_{ij}=1|sex_{ij}=k)}=\alpha +\beta_{1}AGE_{ij}+\beta_{2}RACE_{ij}+\beta_{3}^{*}YEAR_{ij}+\delta_{i}$$

Geospatial model:

$$\log\frac{\mathrm{Pr}(Y_{ij}=1|sex_{ij}=k)}{1-\mathrm{Pr}(Y_{ij}=1|sex_{ij}=k)}=\alpha +\beta_{1}AGE_{ij}+\beta_{2}RACE_{ij}+\beta_{3}^{*}YEAR_{ij}+\beta_{4}{\bar{\delta }}_{i}^{post}+\delta_{i}$$

Covariate model:

$$\log\frac{\mathrm{Pr}(Y_{ij}=1|sex_{ij}=k)}{1-\mathrm{Pr}(Y_{ij}=1|sex_{ij}=k)}=\alpha +\beta_{1}AGE_{ij}+\beta_{2}RACE_{ij}+\beta_{3}^{*}YEAR_{ij}+Z_{i}\gamma +\delta_{i}$$

Full model:

$$\log\frac{\mathrm{Pr}(Y_{ij}=1|sex_{ij}=k)}{1-\mathrm{Pr}(Y_{ij}=1|sex_{ij}=k)}=\alpha +\beta_{1}AGE_{ij}+\beta_{2}RACE_{ij}+\beta_{3}^{*}YEAR_{ij}+Z_{i}\gamma +\beta_{4}{\bar{\delta }}_{i}^{post}+\delta_{i}$$

The person-level predictions of the outcome Y can be aggregated to county prevalence rates for men and women using the US age and county race/ethnic composition for each sex. That is, we estimated the likelihood of having the outcome of interest for one person in each age and race/ethnic group and aggregated these using the respective proportion of people in the age and race/ethnic group for the given sex and the prediction year as weights.

### Test Case: Type 2 Diabetes Prevalence

The outcome variable of interest -- Type 2 diabetes -- was defined as 1 if the respondent answered "yes" to the question: "Have you ever been told by a doctor that you have diabetes?" and zero otherwise. Pregnancy-related temporary diabetes was also coded as zero. Missing, refused, or don't know answers were excluded from the analysis.

Thus, the logit of the probability of a person *j *in county *i *of sex *k *to have Type 2 diabetes (*Y*_*ij *_*= 1) *is assumed to be a linear function of the person's age (*AGE*_*ij*_) and race (*RACE*_*ij*_), survey year (*YEAR*_*ij*_), county-level covariates *Z*_*i *_for the Covariate and Full models of county educational achievement at high school and college degree levels, county poverty rate and median annual household income adjusted for inflation, the number of fast food restaurants per 100,000 population, and the number of medical doctors and dentists per 1,000 population. All models include the county random intercept (*δ*_*i*_). The Geospatial and Full models also include the spatial component in the form of the averaged estimated county random intercept from the Naive and Covariate models, respectively, for neighboring counties (${\bar{\delta }}_{i}^{post}$).

An individual's age and race/ethnicity are potential predictors of a person's probability of having diabetes [11]. In addition, the county's general racial and ethnic composition is assumed to explain differences in diabetes prevalence beyond individual race/ethnicity and in conjunction with other sociodemographic covariates in the model. Modeling time as a fixed effect imposes the same temporal trend on all counties. This assumption may not hold because trends in risk may go up in some counties while staying flat or declining in others. Therefore, we also tested the models with random coefficients on time.

All models were implemented in the R statistical computing language, version 2.10.2 [17].

### Data

Individual age and race/ethnicity information was extracted from the BRFSS and categorized into 10 five-year age groups beginning with 30-34, 35-39, and so forth up to 75+. The group of 50- to 54-year-olds was the largest group and was selected as the reference category. Race was grouped into five mutually exclusive and exhaustive race/ethnicity categories consisting of (i) White non-Hispanic (reference group), (ii) African American and Black non-Hispanic, (iii) American Indian and Alaska Native non-Hispanic, (iv) Hispanic origin, and (v) non-Hispanic Asian, Hawaiian Native and Pacific Islander, other race, multiple or no preferred race. Available counties and sample sizes by year and sex are shown in Table 2, and summary statistics of the covariates are shown in Table 3.

**Table 2:** The number of counties and sample sizes by survey year in the final dataset for persons aged 30 years and older.

Survey Year	Counties in dataset		Sample size in dataset	
	
	Men	Women	Men	Women
1996	2,602	2,768	39,803	58,075
1997	2,698	2,811	43,750	63,420
1998	2,844	2,948	47,680	70,066
1999	2,834	2,959	50,852	75,095
2000	2,907	3,009	58,127	86,386
2001	2,955	3,016	66,065	97,325
2002	2,994	3,050	78,982	119,248
2003	3,007	3,045	84,819	130,777
2004	3,009	3,058	97,198	154,224
2005	3,018	3,064	115,878	186,306
2006	2,747	2,782	111,998	182,346
2007	2,762	2,792	134,325	225,950
2008	2,733	2,765	130,997	218,611

Total	3,140	3,140	1,060,474	1,667,829

**Table 3:** Summary statistics for individual- and county-level covariates.

Variable	Summary Statistic	1996-2004		2000-2008	
		
		Men	Women	Men	Women
Age (years)	Minimum	30	30	30	30
	Mean	50.2	52.3	50.6	52.5
	Maximum	99	99	99	99

Individual Race/Ethnicity (percent)	White	76.6	76.5	73.7	74.2
	Afr. American/Black	8.4	10.0	8.6	9.9
	Asian	4.2	3.3	5.4	4.3
	AIAN	1.0	0.9	1.1	0.9
	Hispanic	9.7	9.3	11.2	10.6

County Race/Ethnicity (percent)	Afr. American/Black	7.8^1^		8.0^1^	
	Hispanic	4.5^1^		5.2^1^	

Education (percent)	High school degree	34.5^2^			
	Bachelor's degree	10.2^2^			

Poverty (percent)	Minimum	1.7^1^		2.6^1^	
	Mean	13.3^1^		13.8^1^	
	Maximum	42.2^1^		39.4^1^	

Household income (thousand U.S. dollars, CPI adjusted)	Minimum	19.0^1^		20.3^1^	
	Mean	45.6^1^		43.4^1^	
	Maximum	114.0^1^		107.9^1^	

Fast food restaurants (number per 100,000 people)	Minimum	5.9^1^		5.3^1^	
	Mean	66.4^1^		71.3^1^	
	Maximum	715.6^1^		1209^1^	

Number of medical doctors per 1,000 population	Minimum	0^1^		0^1^	
	Mean	27.6^1^		27.7^1^	
	Maximum	369.9^1^		370.0^1^	

Number of dentists per 1,000 population	Minimum	0^1^		0^1^	
	Mean	31.9^1^		31.7^1^	
	Maximum	396.1^1^		396.2^1^	

**Note**: Statistics are shown for the periods 1996-2004 and 2000-2008. Figures are adjusted by BRFSS poststratification weight.

^1^Refer to midpoint of the time period, i.e., 2000 for 1996-2004 and 2004 for 2000-2008.

^2^Figures are based on 2000 Census data on educational achievement.

The county-level race/ethnicity composition in the form of the fraction of African Americans/Blacks and the fraction of Hispanics was obtained from the NCHS Bridged-Race population estimates vintage 2008 for 1996-2008. The educational attainment variables were calculated as the fraction of the county population that completed high school and the fraction with bachelor's degrees using 2000 Census data. Median annual household income and the county poverty rate were obtained from the Census Bureau's Small Area Income and Poverty Estimates for 1996-2008. The number of fast food restaurants per 100,000 population was derived from the Census Bureau's County Business Patterns for 1996-2007, the 2008 CBP data were released in July 2010 and could not be considered for this paper, using SIC code 5812 and NAICS code 72221. The transition from SIC to NAICS as well as NAICS revisions that took place in the time period 1999-2007 affected the comparability of the selected SIC and NAICS codes, and the bridge from SIC to NAICS is not fully closed To remove the structural breaks in the time series, the county medians were subtracted from both series, and missing fast food data were multiply imputed using a time series cross-sectional model with auxiliary information and weak priors. The number of medical doctors and dentists was obtained from the Area Resource Files for 1996-2008. Unit- and area-level covariates and outcome variables were linked by unique county five-digit FIPS codes [18,19].

### Creating a Validation Framework

To evaluate the validity of each model, a gold standard of the outcome variable is required. The gold standard serves as a benchmark judged to be the best available direct estimate for the small area domain, i.e., counties with sufficiently large sample sizes, which can be obtained by (i) choosing small domains with large sample sizes in a single survey year, (ii) pooling multiple survey years, or (iii) increasing domain size. We pooled the 2000-2008 BRFSS and calculated the direct age-standardized, sex-specific BRFSS estimates for Type 2 diabetes prevalence, taking poststratification weights into account and weighting each survey year equally. We then used as our gold standard the direct, age-standardized, sex-specific estimates for counties that had more than 900 observations (by sex) in both periods 1996-2004 and 2000-2008. The minimum sample size specified for the gold standard resulted in 121 counties for men and 196 counties for women, and we term these sets the **validation sets**.

The second step of our validation framework involves determining the minimum sample size needed to achieve sufficient correspondence with the gold standard. For this purpose, we sampled with replacement from the available validation set data to obtain 100, 50, and 10 observations per county-year before fitting the model. That is, we systematically reduced the amount of data and information available from the counties with large sample sizes. For other applications, appropriate sample sizes can easily be specified. The sampling process was repeated 10 times at each sampling level so that the average effect of reduced sample size on model validity can be estimated reliably.

We then ran the 16 models for each sex on the 1996-2004 BRFSS and estimated county Type 2 diabetes prevalence rates for 2004. The results were compared against the gold standard for 2004 for the validation set using two metrics:

• Concordance correlation coefficient (CCC), which measures agreement between two variables and correspondence within groups, i.e., how strongly do units within the same county resemble each other.

• Root Mean Squared Error (RMSE) as a measure for the average squared difference between model estimates and the gold standard.

The best-performing models for men and women, respectively, were the ones with the highest CCC and lowest RMSE. They were used in the last step to produce estimates of county Type 2 diabetes prevalence rates in 2008 using 2000-2008 BRFSS data.

### Uncertainty Bounds

We used the best-performing model and calculated empirical 95% credibility regions, obtained by drawing 1,000 samples of the model parameters from their conditional distributions and using them to generate 1,000 sets of individual probabilities of Type 2 diabetes for one person in each county and each age-race group (or age group if individual race was not in the model). We aggregated those to age-standardized and race-specific county prevalence rates using the 2000 US age distribution for the universe of 30+-year-olds and the county-specific race composition. From the 1,000 age-standardized and race-specific Type 2 diabetes prevalence rates, we used the empirical 2.5% and 97.5% values as the bounds for the credibility regions.

## Results

### Identifying the best model for estimating Type 2 diabetes prevalence in the validation framework

**Figure 1:**
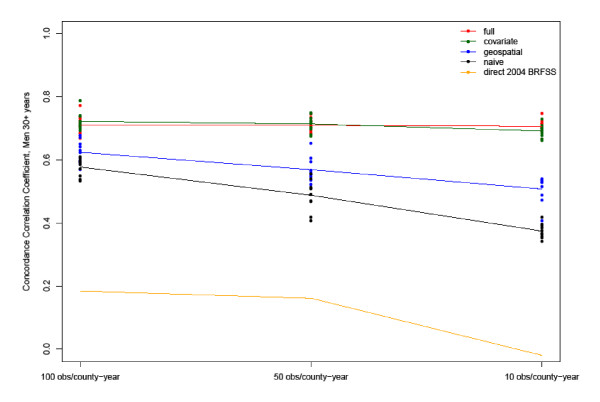
Concordance Correlation Coefficients for the estimates in 2004 of Type 2 diabetes prevalence in men aged 30 years and older from the best-performing model and the gold standard using counties with at least 900 male respondents in the 1996-2004 BRFSS.

**Figure 2:**
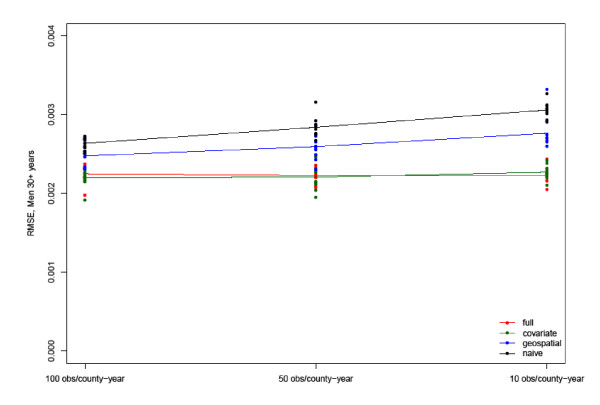
Root Mean Squared Error for the estimates in 2004 of Type 2 diabetes prevalence in men aged 30 years and older from the best-performing model and the gold standard using counties with at least 900 male respondents in the 1996-2004 BRFSS.

Figures 1 and 2 show the CCC and RMSE, respectively, for the best-fitting model for estimated Type 2 diabetes prevalence in 2004 among men 30 years and older. Shown are the CCC and RMSE at the three sampling levels of 100 respondents per county-year (equivalent to a sample size of 900 because we pooled nine years of BRFSS data), 50 respondents per county-year (equivalent to a sample size of 450), and 10 respondents per county-year (equivalent to a sample size of 90). The markers represent the values of the validation metrics for 10 model runs, and the coloring represents the four model families. When the total available sample size is used, the CCC increases to 0.83 for men and 0.85 for women in the full model, respectively (see Additional file 1: Tables S1 and S2). For comparison, we also show the correlation of the gold standard with the single-year direct 2004 BRFSS estimate, which is substantially lower at 0.18 for men and 0.33 for women at the highest sample size and declines further as sample size goes down. The figures look very similar for women (see Additional file 1: Figures S1 and S2).

As illustrated in these figures, the best models for men according to our two metrics are the Full and Covariate models with an individual race covariate included and fixed effects on survey year for both men and women. Compared to the Naïve and Geospatial models, the inclusion of additional relevant covariates significantly improves model fit, especially at very small sample sizes of 10 per county-year. Variations in CCC and RMSE also increase at smaller sample sizes for the Naïve and Geospatial models, illustrating the increasingly weakened ability to accurately estimate the outcome variable for small areas from pure measurement models. Although the Geospatial model still picks up some spatial pattern at small sample sizes compared to the Naïve model, overall CCCs drop from initial values of about 0.82 to between 0.57 and 0.16 at sample sizes of 50 and 10 per county-year, while they remain, on average, at 0.7 for the Covariate and Full models - although individual race emerges as an important explanatory variable in the Naïve and Geospatial models. The superiority of all four models over the direct 2004 BRFSS estimate is dramatic. At sample sizes of 10 per county-year, the CCC drops to near zero for the 2004 BRFSS, and even when the full sample is utilized, it does not exceed 0.43 for men. Adding meaningful covariates can be as effective as increasing the sample sizes five- to tenfold in the Naïve and Geospatial models. This raises further questions regarding the accuracy of single-year, direct survey estimates: Any increase in sample size helps improve precision, but including relevant covariates helps even more as sample sizes become very small.

### Results for the Best Models

**Figure 3:**
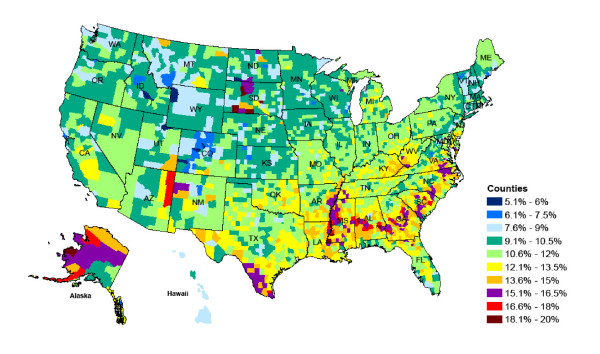
Diabetes prevalence for men in 2008 aged 30 years and older.

**Figure 4:**
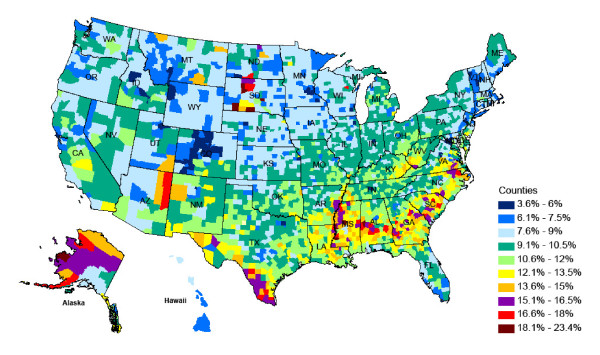
Diabetes prevalence for women in 2008 aged 30 years and older.

The maps in Figures 3 and 4 show the magnitude and distribution of diabetes prevalence for all US counties as estimated from the best-performing model for men and women in 2008.

Men have, on average, slightly higher diabetes prevalence than women, although the highest levels are observed for women. The geographical distribution of high prevalence areas is notable and similar for men and women. High-risk areas are concentrated along the Mexican border in Texas and the southern states of Louisiana, Missouri, Mississippi, and Georgia, North and South Carolina, and southern parts of Virginia. These areas traditionally have higher shares of African Americans/Blacks and Hispanics, who have significant disparities in health status, in part because of lower income and education levels and other sociodemographic characteristics.

Parts of South Dakota, Arizona, and New Mexico that include Native American reservations also have comparatively high rates of diabetes. In contrast, prevalence is lowest in Colorado, Montana, and Wyoming. It is likely that the demographic makeup of the population coupled with lifestyle characteristics play a role in the low diabetes rates of 3.6% to 9%, compared with the national averages of 8.8% for men and 8.2% for women.

The regression coefficient estimates for the best model for men and women, respectively, are shown in Table 4. They are similar, and we observe an upward trend over time, affirming the findings of steadily increasing adult-onset diabetes prevalence [20,21]. Other strong predictors include age, race/ethnicity, and survey year. Diabetes risk more than doubles for men and women from ages 30-34 to 70-74. All race/ethnic groups are estimated to have higher risks of diabetes than Whites, with African Americans/Blacks and American Indians and Alaska Natives experiencing the highest risks of 0.69 and 0.65 for men, respectively, and 0.98 and 0.93 for women, respectively. At the county level, educational achievement is positively correlated with lower diabetes prevalence, especially for women. We also find a strong spatial correlation in Type 2 diabetes prevalence.

**Table 4:** Summary of regression results for estimating Type 2 diabetes prevalence for men and women aged 30 and older for the full model.

Variable	Men			Women		
	
	Estimate		St. error	Estimate		St. error
Intercept	-2.49	***	0.09	-2.36	***	0.08

Age group 30-34	-1.77	***	0.03	-1.52	***	0.02
Age group 35-39	-1.25	***	0.02	-1.12	***	0.02
Age group 40-44	-0.81	***	0.02	-0.75	***	0.02
Age group 45-49	-0.40	***	0.02	-0.39	***	0.01
Age group 55-59	0.39	***	0.01	0.35	***	0.01
Age group 60-64	0.66	***	0.01	0.59	***	0.01
Age group 65-69	0.81	***	0.01	0.75	***	0.01
Age group 70-74	0.91	***	0.01	0.80	***	0.01
Age group 75+	0.80	***	0.01	0.68	***	0.01

African American/Blacks	0.69	***	0.01	0.98	***	0.01
Asian ^§^	0.41	***	0.02	0.51	***	0.02
AIAN	0.65	***	0.03	0.93	***	0.02
Hispanic	0.56	***	0.02	0.75	***	0.01

Year 2001	0.08	***	0.02	0.06	***	0.02
Year 2002	0.11	***	0.02	0.11	***	0.02
Year 2003	0.17	***	0.02	0.16	***	0.02
Year 2004	0.16	***	0.02	0.15	***	0.02
Year 2005	0.23	***	0.02	0.20	***	0.02
Year 2006	0.29	***	0.02	0.23	***	0.02
Year 2007	0.32	***	0.02	0.30	***	0.02
Year 2008	0.34	***	0.02	0.29	***	0.02

Share of African American/Blacks	-0.04		0.05	-0.16	***	0.04
Share of Hispanics	-0.32	***	0.06	-0.37	***	0.04
Share with High school degree	0.19		0.14	-0.09		0.11
Share with Bachelor's degree	-2.14	***	0.21	-3.02	***	0.18
Median annual household income	0.00	*	0.00	0.00		0.00
County poverty rate	0.01	***	0.00	0.01	***	0.00
Fast food restaurants per 100,000 pop.	0.00		0.00	0.00		0.00
Number of medical doctors per 1,000 pop.	0.00	***	0.00	0.00	***	0.00
Number of dentists per 1,000 population	0.00		0.00	0.00		0.00

Spatially averaged random intercept	1.58	***	0.15	2.26	***	0.13
Standard deviation of random intercept			0.098			0.077

Number of counties	3,140			3,140		

Reference race/ethnicity and age group are 50- to 54-year-old Whites. Reference year is 2000. ^§ ^includes Hawaiian Natives and Pacific Islanders, Other race, Multiple race, and No preferred race.***p-value < 0.0001, **p-value < 0.001, *p-value < 0.01, ^^^p-value < 0.05.

#### Uncertainty intervals

**Figure 5:**
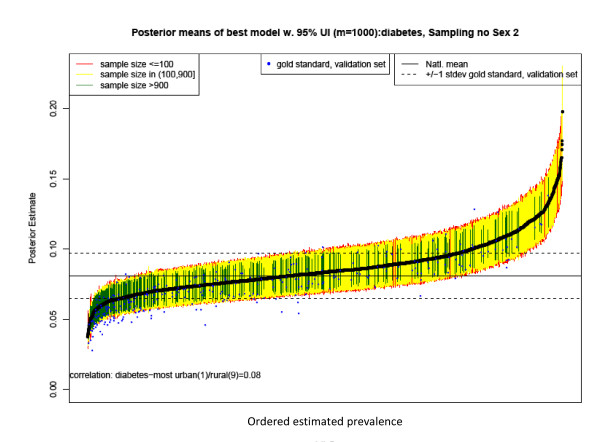
**County estimates and 95% confidence intervals for estimates of Type 2 diabetes prevalence in 2008 for women aged 30 years and older by county**. Note: Intervals are colored according to sample size, with green corresponding to counties with more than 900 observations in 2000-2008, yellow for counties with more than 100 observations, and red for counties with 100 or fewer observations per county-year. The solid black line indicates the national average for women, and the dashed lines represent a standard deviation from the national average for the validation set for women. The correlation is between the estimated diabetes prevalence and the US Department of Agriculture's Urban-Rural Continuum code for 2003 with categorical values ranging from 1 for most urban to 9 for most rural. See http://www.ers.usda.gov/briefing/rurality/ruralurbcon/ for exact definitions.

The 95% empirical credibility regions for women are shown in Figure 5. Counties with large sample sizes have the tightest intervals, and on average, interval length increases as the sample size decreases for the large counties. The majority of counties fall above the national prevalence for Type 2 diabetes of 8.2% for women.

**Figure 6:**
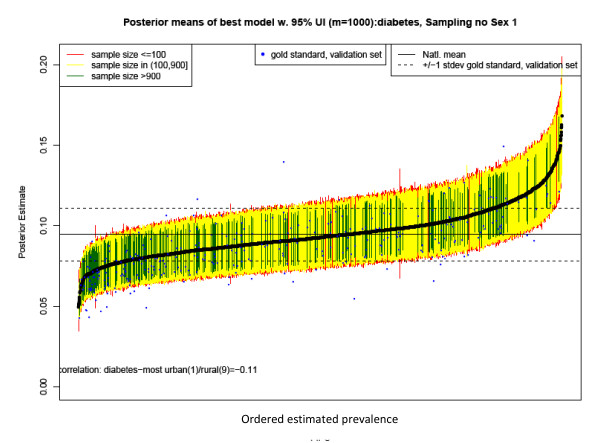
**County estimates and 95% confidence intervals for estimates of Type 2 diabetes prevalence in 2008 for men aged 30 years and older by county**. Note: Intervals are colored according to sample size, with green corresponding to counties with more than 900 observations in 2000-2008, yellow for counties with more than 100 observations, and red for counties with 100 or fewer observations per county-year. The solid black line indicates the national average for men, and the dashed lines represent a standard deviation from the national average for the validation set for men. The correlation is between the estimated diabetes prevalence and the US Department of Agriculture's Urban-Rural Continuum code for 2003 with categorical values ranging from 1 for most urban to 9 for most rural. See http://www.ers.usda.gov/briefing/rurality/ruralurbcon/ for exact definitions.

The pattern is very similar for men, as shown in Figure 6, except for a slightly higher national prevalence of 8.8% and wider confidence intervals due to smaller sample size.

## Discussion and Conclusions

We presented a novel and generalizable methodology for small area measurement and formal out-of-sample validation. Our validation step also provides guidance on the minimum sample size required for future data collection to ensure an accurate estimate of risk factors at the local area. We demonstrated that our methodology can yield more accurate estimates of important health outcomes and risk factors at the local level than single-year, direct survey estimates or pure measurement models. Having validated and local estimates available can help draw attention to health determinants and stimulate research and interventions.

### Limitations

We have used the BRFSS to develop county measurements of Type 2 diabetes prevalence by restricting the population universe to those aged 30 and older. The survey's limitations need to be taken into account when using or interpreting our results. The BRFSS is a telephone survey, and results may be subject to self-reporting bias, although for diabetes, this bias may vary by sex and age and is generally estimated to be relatively small, with estimates comparable to those from NHANES and NHIS [22]. The outcome variable also only measured diagnosed Type 2 diabetes and is therefore likely an underestimate of true Type 2 diabetes prevalence in the US 30+-year-old population.

A second limitation of using the BRFSS arises from the survey's exclusive use of households with landline telephone service. The BRFSS excludes households with no telephone and cellphone-only services. However, BRFSS data consistently provide valid and reliable data when compared to household surveys in the US [23,24]. Moreover, the BRFSS is the only national source of local data in the US.

In the present study, we only tested systematically for spatial patterns for neighboring counties by averaging residual spatial patterns across adjacent counties, i.e., counties that have a common border. That is, we equated adjacency with being more similar than nonadjacent counties. This approach could be expanded in the future by taking topological and other barriers into account and also by considering similarity in feature space, such as sociodemographic characteristics, urbanicity, and other relevant factors.

Our framework hinges on the availability of large domains for which reasonably accurate gold standards can be computed. In our test case, the number of counties with more than 900 respondents in the pooled dataset was 121 (3.9% of all counties) for men and 196 (6.2% of all counties) for women. These are relatively small numbers and can be tested for robustness using different cutoffs for selecting the large domains. We further assume that there is no systematic relationship between domain size and prevalence, a reasonable assumption in our models because the validation counties represent a variety of urban and rural, sociodemographic, and other characteristics.

With respect to our modeling approach, future research will include the examination of models with different variance-covariance structure in the random effects; for example, the explicit modeling of spatial relationships in addition to or in lieu of the spatially pooled residual county random intercept. Current software limitations also limited our ability to incorporate the BRFSS's stratified sampling design. We did, however, use the poststratification weights reported in the BRFSS to calculate direct estimates. Finally, the CCC and RMSE are only two relevant metrics for judging the validity of the model estimates against the gold standard. Other options exist; for example, the ratio of RMSE over the mean for studying the relative size of estimation error.

### Applicability to other settings and as a policy tool

In this paper, we have demonstrated how a validation environment can be created when a subset of small areas in a country have larger samples available. Such a validation environment allows the selection of a modeling strategy that optimally mixes the three approaches of pooling data across time, harnessing spatial patterns in the distribution of the outcome of interest, and adjusting for estimates for local area characteristics. The result is more accurate and precise small area measurements. We believe the approach that we have outlined can be applied in a straightforward manner to a full range of variables collected in surveys such as the BRFSS to generate annual measurements at the county level for a wide array of health behaviors and service utilization. These local and annual measurements can be an important stimulus to local public health decision-making and community engagement.

Another implication of the small area validation study demonstrated here is that samples as small as 50 observations per county and year can - with the appropriate analytical tools - yield quite robust measurements with acceptable uncertainty intervals. In contrast, the current practice used in many states and policy analyses of using small samples in statistical analyses can result in estimates with very low correlations with de facto gold standards based on large samples. Many counties in the United States have conducted their own BRFSS surveys. However, due to the considerable costs of such surveys, data collection is not carried out on a yearly basis. Our framework provides an affordable strategy for such data collection. Local health departments could contract with the BRFSS to ensure minimum sample sizes of approximately 50 respondents per year at a much lower additional cost.

The test case of Type 2 diabetes demonstrated that while the US is generally data-rich, it also lacks accurate, timely information on status and trends in leading health risks. In a US context, our methodology could be used to produce local estimates of the leading risk factors for the US burden of disease that enable local and state health officials to prioritize and target high-risk counties while spending local, state, and federal funds more wisely on prevention and treatment programs. The generation of local health outcome and risk factor estimates over time will also allow the tracking of progress to first slow and then reverse trends in major risk factors. Being able to compare county efforts to reduce the prevalence of diabetes or other diseases on a dollar-spent-per-point-reduction basis would create positive competition and allow identification of best practices.

In addition to health status and risk factor analysis in resource-rich countries such as the US, the framework can easily be applied to countries with large but locally insufficient health surveys and administrative databases. It can also be extended to obtain coverage estimates of important health interventions. For low-income, resource-scarce countries, it is particularly attractive to use existing administrative and survey data to get more accurate local coverage estimates as it allows the identification of "hot spots" and more efficient and effective targeting of interventions. For example, our methodology could be used in resource-poor countries with large Demographic Health Surveys to produce local estimates of health risk factors and diseases.

Our framework pushes open the door to more systematically, accurately, and efficiently use available data to track the status and effects of public policy interventions. It allows public health professionals to obtain accurate estimates of major health outcomes and risk factors and therefore to design and implement adequate preventive measures to reduce the burden of disease. Our methodology could be used to track progress and allocate resources to improve health at the local level.

## Competing interests

The authors declare that they have no competing interests.

## Authors' contributions

CJLM conceived of the study's idea, helped with the analysis, and contributed to writing the manuscript. TS carried out the data compilation, modeling, and statistical analysis, and wrote the first draft of the manuscript. AHM participated in the design of the study, data collection, and edited the manuscript. All authors read and approved the final manuscript.

## Supplementary Material

Additional file 1 **Figure S1**: Concordance Correlation Coefficients for model validation for Type 2 diabetes prevalence in women aged 30 years and older in 2004 using counties with at least 900 female respondents in the 1996-2004 BRFSS. The file contains a 5-colored graphic of the concordance correlation coefficient showing how well the four model families and the direct, single year survey estimate correlate with the gold standard for diabetes prevalence in 30+ year old women. Figure S2: Root Mean Squared Error for model validation for Type 2 diabetes prevalence in women aged 30 years and older in 2004 using counties with at least 900 female respondents in the 1996-2004 BRFSS. The file contains a 4-colored graphic of the root mean squared error showing how the square root of the average squared deviation of the estimated from the four model families from the gold standard for diabetes prevalence in 30+ year old women. Table S1: Concordance correlation coefficients for the estimated Type 2 diabetes prevalence in 2004 in men 30 years and older and the gold standard using counties with at least 900 observations in the pooled 1996-2004 BRFSS. The file contains a table summarizing the concordance correlation coefficient for each model family, model specification, and sampling level with the gold standard in 30+ year old men. Table S2: Concordance correlation coefficients for the estimated Type 2 diabetes prevalence in 2004 in women 30 years and older and the gold standard using counties with at least 900 observations in the pooled 1996-2004 BRFSS. The file contains a table summarizing the concordance correlation coefficient for each model family, model specification, and sampling level with the gold standard in 30+ year old women. Click here for file
