# Supplementary material for: A novel framework for validating and applying standardized small area measurement strategies
Source: Popul Health Metr. 2010 Sep 29;8:26. doi: 10.1186/1478-7954-8-26 (PMC2958154; doi:10.1186/1478-7954-8-26)
Supplement: Additional file 1 — Figure S1: Concordance Correlation Coefficients for model validation for Type 2 diabetes prevalence in women aged 30 years and older in 2004 using counties with at least 900 female respondents in the 1996-2004 BRFSS. The file contains a 5-colored graphic of the concordance correlation coefficient showing how well the four model families and the direct, single year survey estimate correlate with the gold standard for diabetes prevalence in 30+ year old women. Figure S2: Root Mean Squared Error for model validation for Type 2 diabetes prevalence in women aged 30 years and older in 2004 using counties with at least 900 female respondents in the 1996-2004 BRFSS. The file contains a 4-colored graphic of the root mean squared error showing how the square root of the average squared deviation of the estimated from the four model families from the gold standard for diabetes prevalence in 30+ year old women. Table S1: Concordance correlation coefficients for the estimated Type 2 diabetes prevalence in 2004 in men 30 years and older and the gold standard using counties with at least 900 observations in the pooled 1996-2004 BRFSS. The file contains a table summarizing the concordance correlation coefficient for each model family, model specification, and sampling level with the gold standard in 30+ year old men. Table S2: Concordance correlation coefficients for the estimated Type 2 diabetes prevalence in 2004 in women 30 years and older and the gold standard using counties with at least 900 observations in the pooled 1996-2004 BRFSS. The file contains a table summarizing the concordance correlation coefficient for each model family, model specification, and sampling level with the gold standard in 30+ year old women. [file 1478-7954-8-26-S1.DOCX]

**Additional Files**

**Figure S1: Concordance Correlation Coefficients for model validation for Type 2 diabetes prevalence in women in 2004 using counties with at least 900 female respondents in the 1996-2004 BRFSS.**

**Figure S2: Root Mean Squared Error for model validation for Type 2 diabetes prevalence in women in 2004 using counties with at least 900 female respondents in the 1996-2004 BRFSS.**

**Table S1: Concordance correlation coefficients for the estimated Type 2 diabetes prevalence in 2004 in men 30 years and older and the gold standard using counties with at least 900 observations in the pooled 1996-2004 BRFSS.**

| Sex | Model Family | Time Covariate | Race Covariate | Using all observations | 100 observations per county year | 50 observations per county year | 10 observations per county year |
| --- | --- | --- | --- | --- | --- | --- | --- |
| Males | Full | FE | out | 0.81 | 0.69 | 0.69 | 0.7 |
|  |  | FE | in | 0.83 | 0.71 | 0.71 | 0.71 |
|  |  | RE | out | 0.74 | 0.51 | 0.49 | 0.43 |
|  |  | RE | in | 0.79 | 0.57 | 0.54 | 0.48 |
|  | Covariate | FE | out | 0.82 | 0.7 | 0.69 | 0.68 |
|  |  | FE | in | 0.84 | 0.72 | 0.71 | 0.69 |
|  |  | RE | out | 0.75 | 0.5 | 0.47 | 0.4 |
|  |  | RE | in | 0.8 | 0.57 | 0.52 | 0.46 |
|  | Geospatial | FE | out | 0.8 | 0.56 | 0.48 | 0.34 |
|  |  | FE | in | 0.82 | 0.62 | 0.57 | 0.51 |
|  |  | RE | out | 0.75 | 0.47 | 0.35 | 0.25 |
|  |  | RE | in | 0.8 | 0.55 | 0.49 | 0.48 |
|  | Naïve | FE | out | 0.8 | 0.51 | 0.39 | 0.13 |
|  |  | FE | in | 0.82 | 0.58 | 0.49 | 0.37 |
|  |  | RE | out | 0.76 | 0.45 | 0.33 | 0.16 |
|  |  | RE | in | 0.81 | 0.54 | 0.48 | 0.44 |

**Table S2: Concordance correlation coefficients for the estimated Type 2 diabetes prevalence in 2004 in men 30 years and older and the gold standard using counties with at least 900 observations in the pooled 1996-2004 BRFSS.**

| Sex | Model Family | Time Covariate | Race Covariate | Using all observations | 100 observations per county year | 50 observations per county year | 10 observations per county year |
| --- | --- | --- | --- | --- | --- | --- | --- |
| Females | Full | FE | out | 0.88 | 0.83 | 0.82 | 0.81 |
|  |  | FE | in | 0.89 | 0.84 | 0.82 | 0.8 |
|  |  | RE | out | 0.87 | 0.77 | 0.74 | 0.7 |
|  |  | RE | in | 0.89 | 0.78 | 0.77 | 0.74 |
|  | Covariate | FE | out | 0.88 | 0.83 | 0.82 | 0.8 |
|  |  | FE | in | 0.88 | 0.83 | 0.81 | 0.78 |
|  |  | RE | out | 0.87 | 0.76 | 0.73 | 0.68 |
|  |  | RE | in | 0.89 | 0.77 | 0.75 | 0.71 |
|  | Geospatial | FE | out | 0.89 | 0.73 | 0.64 | 0.44 |
|  |  | FE | in | 0.9 | 0.77 | 0.7 | 0.59 |
|  |  | RE | out | 0.86 | 0.67 | 0.6 | 0.4 |
|  |  | RE | in | 0.89 | 0.74 | 0.71 | 0.69 |
|  | Naïve | FE | out | 0.89 | 0.7 | 0.59 | 0.28 |
|  |  | FE | in | 0.9 | 0.73 | 0.64 | 0.49 |
|  |  | RE | out | 0.87 | 0.68 | 0.59 | 0.29 |
|  |  | RE | in | 0.9 | 0.72 | 0.68 | 0.63 |
